# Impact of catheter tip to hepatic vein ostium distance on the validity and prognostication of hepatic venous pressure gradient in cirrhosis

**DOI:** 10.1038/s41598-023-44016-7

**Published:** 2023-10-09

**Authors:** Hiang Keat Tan, Alfred Bingchao Tan, Kevin Kim Jun Teh, Apoorva Gogna, Chow Wei Too, Sum Leong, Jason Pik Eu Chang

**Affiliations:** 1 Department of Gastroenterology and Hepatology, Singapore General Hospital, Singapore, Singapore; 2 Department of Vascular and Interventional Radiology, Singapore General Hospital, Singapore, Singapore

**Keywords:** Liver cirrhosis, Portal hypertension, Hepatic portal vein

## Abstract

Hepatic venous pressure gradient (HVPG) is an accurate measure of portal hypertension in cirrhosis. However, the effect of catheter tip distance from hepatic vein ostium (HVO) on HVPG is unknown. We performed a retrospective study on 228 patients with 307 HVPGs in our institution. The objectives of this study were to assess the effect of catheter position on the validity of HVPG and its prognostication in cirrhosis. In this study, free hepatic vein pressure (FHVP) was considered optimal when difference between FHVP and inferior vena cava pressure was ≤ 2 mmHg. HVPG progressively decreased (*p* < 0.001) when measured at increasing distance from HVO due to an increasing FHVP (*p* = 0.036) but an unchanged wedged hepatic vein pressure (*p* = 0.343). Catheter tip distance > 5 to ≤ 8 cm [odds ratio {OR} 0.16 (95% CI 0.05–0.47), *p* = 0.001] and > 8 cm [OR 0.14 (95% CI 0.04–0.47), *p* = 0.002] compared to ≤ 3 cm from HVO were independent predictors of not achieving optimal FHVP. Baseline HVPG ≥ 16 mmHg was strongly associated with deaths due to cirrhosis and liver transplantation for end-stage liver disease compared to HVPG < 16 mmHg when FHVP was optimal (*p* < 0.001) but not when it was suboptimal (*p* = 0.359). Our study showed that FHVP is spuriously elevated when measured at > 5 cm from HVO, resulting in inaccurately low HVPG.

## Introduction

“Anything worth doing should be done right”. Groszmann and Wongcharatrawee^1^ were the first to describe the quality indicators for accurate and reliable measurement of hepatic venous pressure gradient (HVPG) in 2004. Although wedged hepatic venous pressure (WHVP) was described as early as 1951 by Taylor and Myers^2^, much of the early research on portal hypertension (PH) relied on the more invasive direct measurement of portal vein pressure^3,4^. It was subsequently shown that that WHVP was highly correlated with the direct portal vein pressure in cirrhosis of varying aetiologies^5–7^, obviating the need for puncture of portal vein for assessment of portal hypertension. In 1979, Groszmann et al.^8^ introduced the use of balloon catheter to achieve wedged position in the hepatic vein (HV) instead of a straight catheter, allowing for repeated measurement of WHVP and free hepatic vein pressure (FHVP) without the need for multiple catheter manipulations.

Despite the extensive body of literature supporting the role of HVPG in clinical management, its use remains largely restricted to centres of academic excellence. Despite this, several groups have continued to refine the techniques of HVPG to improve its value as a prognostic tool^9–11^. Central to the calculation of HVPG is the accurate measurement of free hepatic vein pressure (FHVP) as a surrogate of systemic pressure. The technique of FHVP measurement remains inconsistent, where some have advocated for the catheter to be placed strictly 2–3 cm from hepatic vein ostium (HVO)^9,10^ while others measured FHVP at either 2–4 or 4–5 cm from HVO^12,13^. It was also suggested that the difference between FHVP and inferior vena cava pressure at the level of hepatic vein (IVCP), or FHVP-IVCP > 2 mmHg, may be clue that FHVP measurements had been too distal^1^ but there is no data supporting this recommendation.

Therefore, the aims of this study were to evaluate (1) the effect of HVPG measurements at various distance from HVO and (2) the relationship of catheter position on FHVP-IVCP and its impact on the accuracy of HVPG in clinical correlation and prognostication in cirrhosis.

## Patients and methods

### Study population

We conducted a clinical audit in 2021 to evaluate the quality of HVPGs performed at Singapore General Hospital, a tertiary hospital with a liver transplant programme. In keeping with national guidelines on quality assurance and/or service improvement studies by the Ministry of Health Singapore^14^, this study did not require ethics board approval and informed patient consent as it was a retrospective review of service provision. Access to individual patient electronic medical records was approved (Ref: MR1811-21/R) in accordance with our institutional policy for governance of data use by the Office of Data and Digital Governance, SingHealth. This involves review and endorsement by the departmental head, institutional data protection officer and chief executive officer.

**Figure 1:**
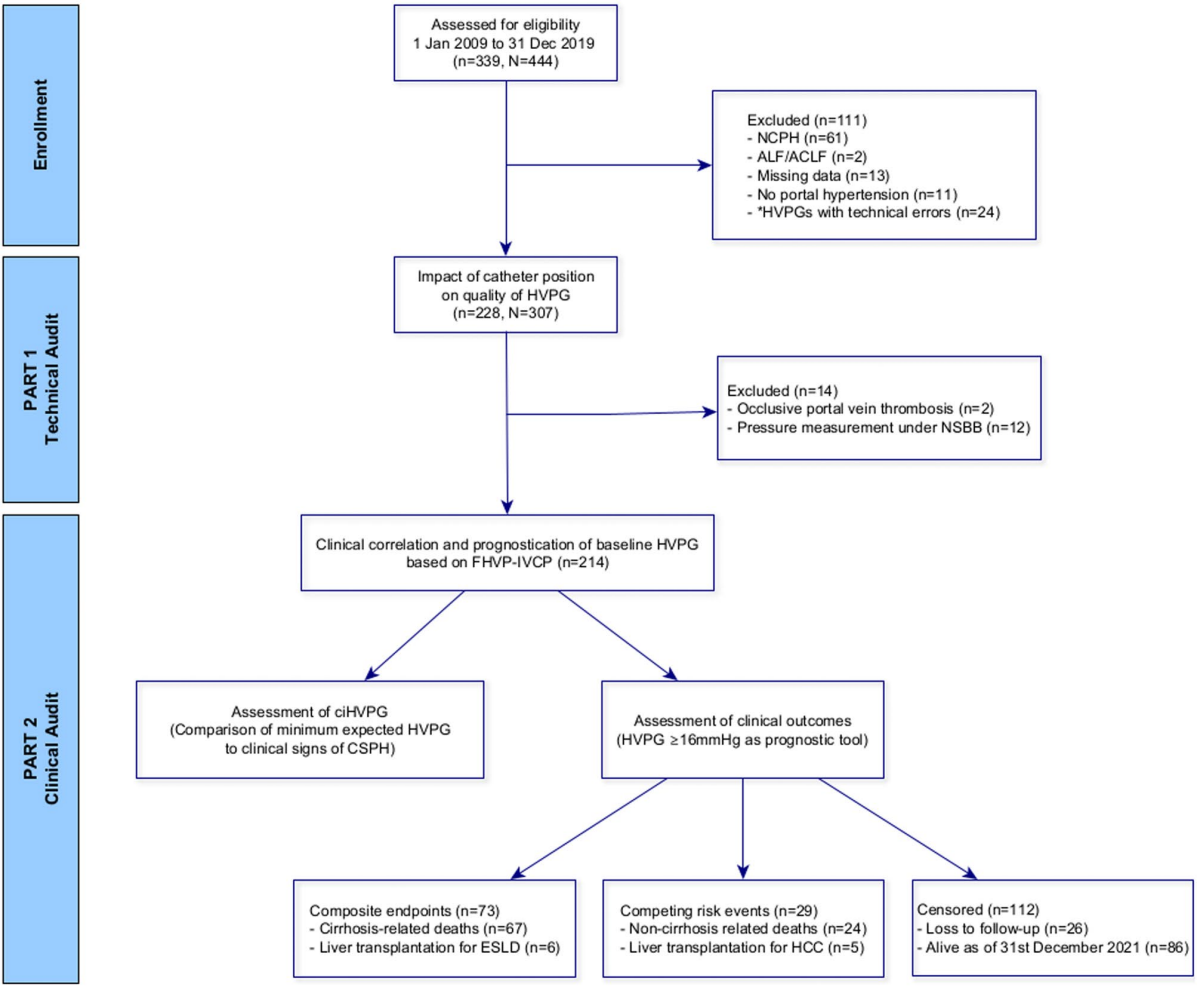
Flow chart of the study. ‘N’ refers to the number of HVPG procedures while ‘n’ refers to the number of patients. ACLF, acute-on-chronic liver failure; ALF, acute liver failure; ciHVPG, clinically incongruent hepatic venous pressure gradient; ESLD, end-stage liver disease; FHVP-IVCP, difference between free hepatic vein pressure and inferior vena cava pressure at the level of hepatic vein; HCC, hepatocellular carcinoma; NCPH, non-cirrhotic portal hypertension; NSBB, non-selective beta-blocker. *HVPGs with technical errors: inconsistencies in the repeat FHVP or WHFP measurements that differed by > 2 mmHg (n = 5), significant intrahepatic veno-venous communications in the hepatic vein (n = 9), inadequate balloon occlusion on venogram (n = 3) procedures with single pressure measurements only (n = 6) and HVPG under deep sedation (n = 1).

All patients with cirrhosis aged ≥ 21 years with HVPG(s) performed between 1 January 2009 and 31 December 2019 were assessed for eligibility. Cirrhosis was diagnosed by histology, or combination of clinical, biochemical, ultrasonographic and/or endoscopic findings. The underlying causes of cirrhosis were identified by clinical history, serology and/or histology^15^ but the diagnosis of nonalcoholic steatohepatitis (NASH) cirrhosis was based on the consensus recommendations by the multi-stakeholder Liver Forum for definite (n = 20) and probable (n = 59) NASH^16^. Briefly, definite NASH required histological confirmation of steatohepatitis while probable NASH was diagnosed in the presence of steatosis without steatohepatitis on histology or steatosis on imaging plus at least two cardiometabolic risk factors and in the absence of competing aetiology. Patients were then assessed for unequivocal signs of clinically significant portal hypertension (CSPH) within three months of each HVPG: gastroesophageal varices, ascites, hepatic hydrothorax and/or abdominal portosystemic collaterals. Exclusion criteria were non-cirrhotic portal hypertension (n = 61), acute liver failure or acute-on-chronic liver failure (n = 2), absence of portal hypertension (HVPG ≤ 5 mmHg and no unequivocal sign of CSPH) (n = 11), HVPGs with technical errors (n = 24) and patients with missing data (n = 13) (Fig. 1).

### Haemodynamic assessment and technical review of HVPG

**Figure 2:**
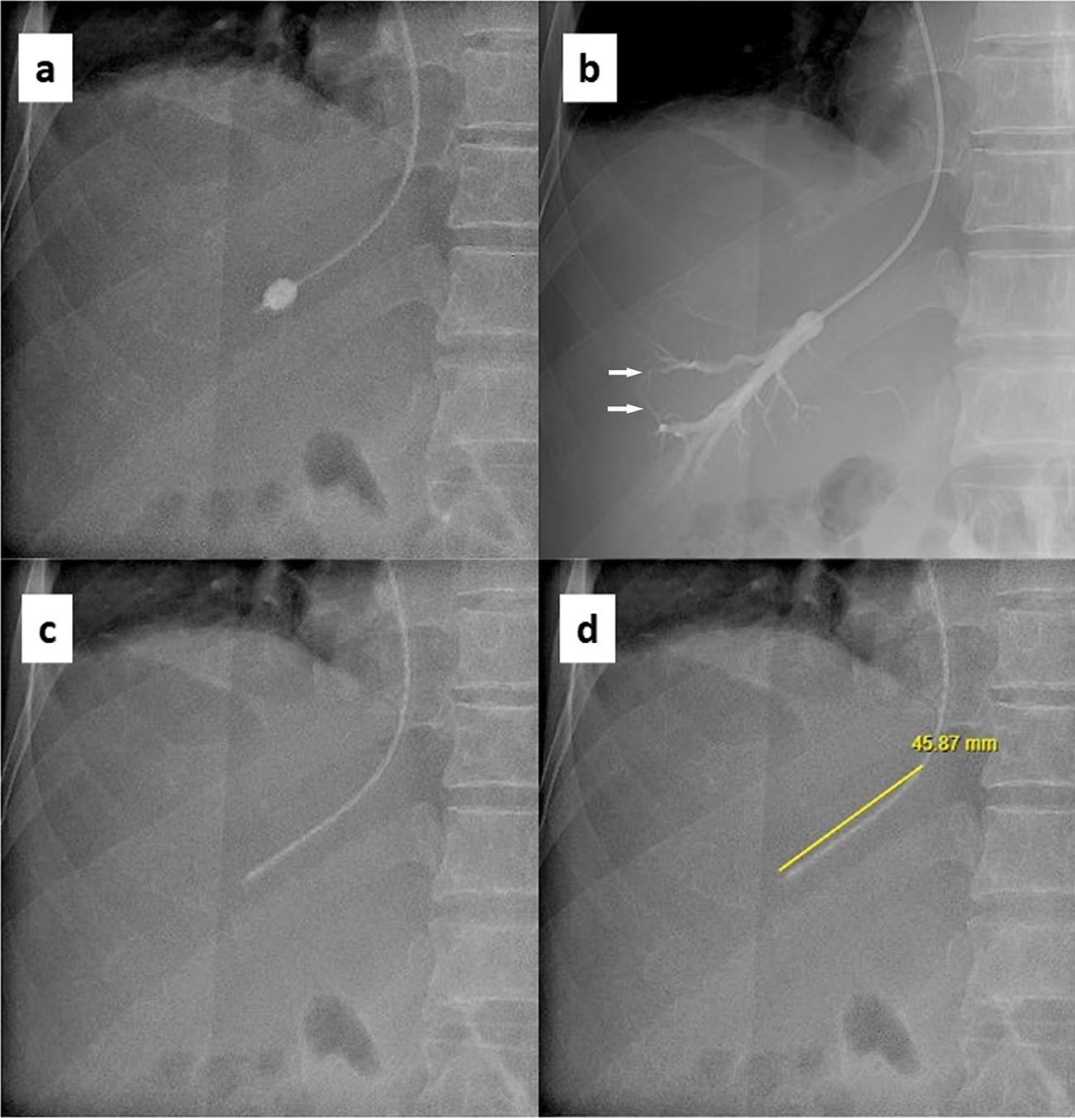
Fluoroscopy images of hepatic venous pressure gradient measurement. (**a**) Catheter position during measurement of wedged hepatic vein pressure. (**b**) Balloon occlusion venography. (**c**) Catheter position during measurement of free hepatic vein pressure. (**d**) Measurement of catheter tip distance from hepatic vein ostium, using the built-in tool on electronic picture archiving and communications system (Vue Motion; Philips, Amsterdam, Netherlands) in our institution. Arrows in panel (**b**) show a tiny veno-venous communication demonstrated on balloon occlusion venogram which was unlikely to have any significant impact on the wedged hepatic vein pressure as the communication was confined to the wedged segment of the hepatic vein and did not communicate with the systemic venous system.

Briefly, all procedures were executed by interventional radiologists via ultrasound-guided transjugular approach under local anaesthesia as previously described^17^. No patient in this study received deep sedation or general anaesthesia during HVPG. Once either the right or middle hepatic vein was cannulated with a 4F Multipurpose A catheter (Cook Medical, Bloomington, IN), a 5.5F balloon-tipped catheter (Fogarty, Edward Lifesciences, Irvine, CA) was exchanged and triplicate pressures were recorded with a digital pressure transducer (IntelliVue MX450, Philips, Amsterdam, Netherlands) in the same position. Wedged hepatic vein pressure (WHVP) was obtained when the balloon was fully inflated (Fig. 2a) to ensure a satisfactorily occluded position and to exclude intrahepatic veno-venous shunts (Fig. 2b) while FHVP was obtained with the catheter freely floating after deflation of the balloon (Fig. 2c). In both pressure measurements, tracings were recorded for 45–60 s until stable readings were obtained. In measurements with FHVP-IVCP > 2 mmHg, repeat hepatic vein and inferior vena cava venography was carried out to exclude focal stenosis. HVPG was calculated as the difference between WHVP and FHVP. In this study, optimal FHVP was defined by FHVP-IVCP ≤ 2 mmHg while suboptimal FHVP was defined by FHVP-IVCP > 2 mmHg as FHVP-IVCP ≤ 2mmHg^1,10,12^. After completion of WHVP and FHVP measurements, the catheter was then placed in the intrahepatic portion of inferior vena cava and right atrium for the measurement of IVCP and right atrial pressure, respectively, in all patients.

For this study, HVPG procedures were assessed by reviewing fluoroscopy images stored digitally in an electronic picture archiving and communications system (Vue Motion; Philips, Amsterdam, Netherlands) which has multiple built-in electronic tools, including for the measurement of length. Catheter tip distance from HVO was measured^12^ (Fig. 2d) by the first author (H.K.T.) who was blinded to other HVPG and clinical outcomes data. To ensure consistency, 10% of the HVPGs were randomly selected for repeat measurements of the catheter tip distance by the first author again and the senior author (J.P.E.C) of this study.

### Study outcomes

The first part of the study was a technical audit of HVPG procedural quality in our institution (Fig. 1). The aim was to assess the impact of catheter position within the hepatic vein on HVPG and to evaluate its relationship to optimal FHVP, which is an important determinant of HVPG validity.

The second part of the study was a clinical audit to evaluate the impact of FHVP-IVCP on clinical correlation and prognostic value of HVPG (Fig. 1). In patients with ≥ 2 HVPGs, only the baseline pressure measurement was included. Patients with non-selective beta-blocker (n = 12) or occlusive portal vein thrombosis (n = 2) at the time of HVPG measurements were excluded.

The baseline HVPG for each patient was compared to the minimum expected HVPG for CSPH. HVPG lower than expected for their respective sign(s) of CSPH were deemed clinically incongruent HVPG (ciHVPG):

-  < 10 mmHg in patients with gastric, low risk oesophageal varices (small varices without red sign)^18^, or ascites/hepatic hydrothorax^19^
-  < 10 mmHg in patients without gastroesophageal varices, ascites or hepatic hydrothorax but with abdominal portosystemic collaterals seen on any abdominal scans^20^
-  < 12 mmHg in patients with non-bleeding, high risk oesophageal varices (small varices with red signs or large varices)^21,22^
-  < 12 mmHg in patients with history of oesophageal variceal bleed within the past three months^23^

Finally, the association of HVPG ≥ 16 mmHg to the composite endpoints of cirrhosis-related death and liver transplantation for end-stage liver disease (ESLD)^24^ was assessed. Deaths were due to cirrhosis if they were directly related to complications of cirrhosis, within six weeks of variceal bleed or following acute-on-chronic liver failure^25^. Patients lost to follow-up were censored at last known date alive while those still on follow up were censored on 31 December 2021. Non-cirrhosis-related deaths and liver transplantation for hepatocellular carcinoma only without clinical decompensation were competing risk events.

### Statistical analysis

Continuous variables were expressed as median and interquartile range (IQR) while categorical variables were expressed as numbers and their percentages in parentheses. Continuous variables were compared by means of the Mann–Whitney U test, while categorical variables by either the Pearson’s χ^2^ or Fisher’s exact test. The intra-class correlation coefficient (ICC) was calculated to determine the degree of rater agreement in the measurement of catheter tip distance from HVO. The Jonckheeree-Terpstra test for trend was performed to compare pressure measurements in ordered groups of increasing catheter tip distance from HVO during HVPG measurement. Post-hoc pairwise comparisons of HVPG measurements with Bonferroni correction were also assessed.

A multivariable binary logistic regression was conducted to assess independent predictors of optimal FHVP and ciHVPG. All variables with *p* < 0.100 on univariate analysis were selected for inclusion into multivariable analysis. Competing risk Gray’s test was applied to compare cumulative incidence of the composite endpoints of patients with HVPG ≥ 16mHg and < 16 mmHg at baseline. Statistical analysis was performed with SPSS v26.0 (IBM Corp., Armonk, NY, USA) and EZR v1.55, a graphical user interface for R software v4.1.2 (https://www.r-project.org)^26^ where a two-sided *p* < 0.050 was statistically significant.

## Results

### Study population

Three-hundred thirty-nine patients underwent 444 HVPGs during the 10-year period. After excluding 111 patients, 228 patients with 307 HVPGs were included in this study (Fig. 1). Table 1 shows baseline characteristics of the study population. Median age of patients was 62.2 (IQR 54.7–68.2) years and there was a predominance of male gender (57.0%). Cirrhosis was mostly due to NASH (34.6%), viral hepatitis (29.4%) and alcoholic liver disease (13.6%). The Child–Pugh and MELD scores were 6 (5–8) and 9 (8–13), respectively. Most patients (n = 213, 93.4%) had one or more sign(s) of CSPH.

**Table 1:** Baseline characteristics of all patients.

Characteristics	All patients (n = 228)
Age, years	62.2 (54.7–68.2)
Gender, n (%)	
Male	130 (57.0)
Female	98 (43.0)
Aetiology of cirrhosis, n (%)	
NASH	79 (34.6)
Viral hepatitis	67 (29.4)
Alcoholic liver disease	31 (13.6)
Autoimmune liver disease	20 (8.8)
Others	17 (7.5)
Unequivocal signs of portal hypertension, n (%)	
Abdominal portosystemic collaterals	124 (54.4)
History of variceal bleed	95 (41.7)
Oesophageal	74 (77.9)
Gastric	16 (16.8)
Ectopic	5 (5.3)
Non-bleeding varices	112 (49.1)
High risk oesophageal	72 (64.3)
Low risk oesophageal	26 (23.2)
Gastric	25 (22.3)
Ascites/Hepatic hydrothorax	82 (36.0)
None	15 (6.6)
Other complications, n (%)	
Occlusive portal vein thrombosis	2 (0.9)
Hepatic encephalopathy	9 (3.9)
Hepatocellular carcinoma	29 (12.8)
Haemoglobin (g/dL)	10.8 (9.4–12.5)
WBC (× 10^9^/L)	4.79 (3.78–6.74)
Platelet (× 10^9^/L)	103 (75–142)
Albumin (g/L)	32 (27–36)
Bilirubin (µmol/L)	21 (15–36)
ALT (U/L)	34 (22–51)
AST (U/L)	49 (37–69)
INR	1.12 (1.04–1.24)
Urea (mmol/L)	4.4 (3.0.0–5.9)
Sodium (mmol/L)	137 (135–139)
Creatinine (µmol/L)	66 (54–83)
MELD score	9 (8–13)
Child–Pugh score	6 (5–8)

Continuous data are presented as median (interquartile range) and categorical data as count (%).

ALP, alkaline phosphatase; ALT, alanine aminotransferase; AST, aspartate aminotransferase; INR, international normalized ratio; MELD, Model for End-Stage Liver Disease score; WBC, white blood cell.

### Impact of catheter position on HVPG measurements

Of the 307 HVPGs, FHVP was optimal in 190 (61.9%) and suboptimal in the remaining 117 (38.1%). The catheter tip distance from HVO was 4.9 (IQR 4.1–6.4, range 1.8–13.0) cm during pressure measurements, where 165 HVPG measurements (53.7%) were obtained at ≤ 5 cm from HVO (Table 2). The intra- and inter-rater ICC for measurement of catheter tip distance from HVO were 0.96 [(95% CI 0.94–0.98), *p* < 0.001] and 0.88 [(95% CI 0.75–0.94), p < 0.001], respectively.

**Table 2:** Hemodynamic characteristics of HVPG measurements.

Characteristics	Total procedures (N = 307 )
WHVP (mmHg)	26 (22–31)
FHVP (mmHg)	11 (8–15)
HVPG (mmHg)	15 (12–18)
IVCP (mmHg)	8 (6–12)
FHVP-IVCP (mmHg) Optimal FHVP, n (%)	2 (IQR 1–4, range 0–17) 190 (61.9)
Catheter tip distance from HVO (cm) ≤ 3 cm, n (%) > 3 to ≤ 5 cm, n (%) > 5 to ≤ 8 cm, n (%) > 8 cm, n (%)	4.9 (IQR 4.1–6.4, range 1.8–13.0) 24 (7.8) 141 (45.9) 104 (33.9) 38 (12.4)

Continuous data are presented as median (interquartile range) and categorical data as count (%).

FHVP, free hepatic vein pressure; FHVP-IVCP, difference between free hepatic vein and inferior vena cava pressure at level of hepatic vein; HVO, hepatic vein ostium; HVPG, hepatic venous pressure gradient; IQR, inter-quartile range; IVCP, inferior vena cava pressure at level of hepatic vein; WHVP, wedged hepatic vein pressure.

**Figure 3:**
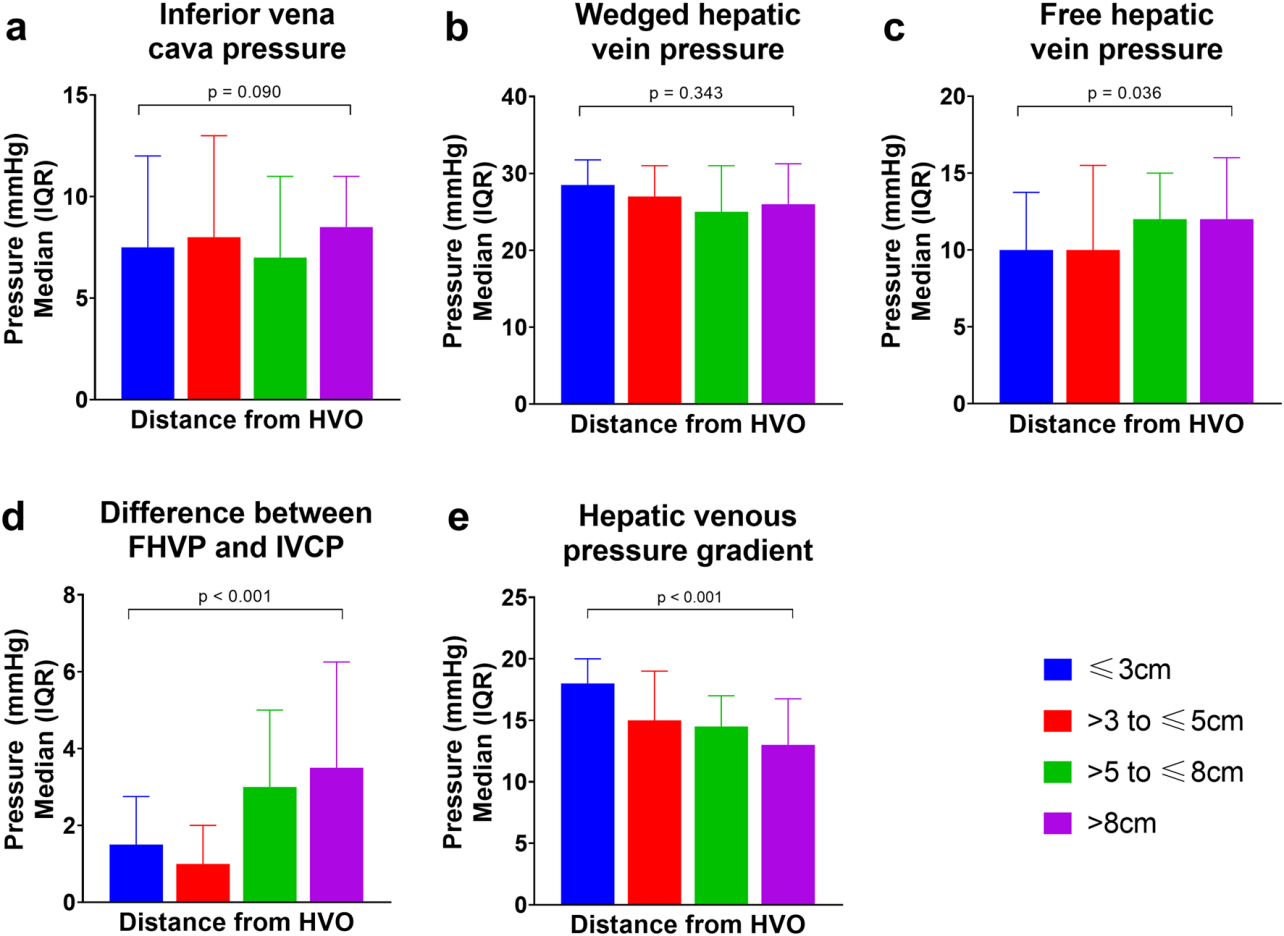
Pressure measurements, grouped according to catheter tip distance from HVO. (**a**) Inferior vena cava pressure. (**b**) Wedged hepatic vein pressure. (**c**) Free hepatic vein pressure. (**d**) Difference between FHVP and IVCP. (**e**) Hepatic venous pressure gradient. FHVP, free hepatic vein pressure; HVO, hepatic vein ostium; IVCP, inferior vena cava pressure at the level of hepatic vein.

Figure 3 depicts the impact of catheter tip distance from HVO on the pressure recordings during HVPG measurements. Although WHVP (*p* = 0.343) (Fig. 3a) and IVCP (*p* = 0.090) (Fig. 3b) are not affected by catheter position, their pressure changes are included in the graph for illustrative purposes. There was a statistically significant trend for a higher FHVP (*p* = 0.036) with increasing catheter tip distance from HVO (Fig. 3c). With the increasing FHVP, there was also a statistically significant trend of a progressively larger FHVP-IVCP (*p* < 0.001) (Fig. 3d). As a result, a decreasing trend of HVPG was observed with a more distal catheter position in the hepatic vein during pressure measurement (*p* < 0.001) (Fig. 3e). Post-hoc pairwise comparisons showed no statistically significant difference in the HVPGs measured at ≤ 3 compared to > 3 to ≤ 5 cm from HVO (*p* = 0.190) and between > 5 to ≤ 8 and > 8 cm from HVO (*p* = 1.000) while all other between-group comparisons were significantly different (Table 3).

**Table 3:** Post-hoc pairwise comparisons of HVPGs measured at different distance from HVO.

Reference HVPG	Comparator HVPG	Unadjusted *p* value	Adjusted *p* value^a^
≤ 3 cm	> 3 to ≤ 5 cm	0.032	0.190
≤ 3 cm	> 5 to ≤ 8 cm	< 0.001	0.003
≤ 3 cm	> 8 cm	0.002	0.011
> 3 to ≤ 5 cm	> 5 to ≤ 8 cm	0.002	0.012
> 3 to ≤ 5 cm	> 8 cm	0.002	0.015
> 5 to ≤ 8 cm	> 8 cm	0.182	1.000

^a^Adjusted by the Bonferroni correction for multiple comparisons.

HVO, hepatic vein ostium; HVPG, hepatic venous pressure gradient.

Table 4 shows the univariate and multivariable binary logistic regression for factors associated with optimal FHVP during HVPG measurement. On multivariable analysis, only catheter tip distance > 5 to ≤ 8 cm [odds ratio {OR} 0.16 (95% CI 0.05–0.47), *p* = 0.001] and > 8 cm [OR 0.14 (95% CI 0.04–0.47), *p* = 0.002] from HVO were independent predictors of not achieving optimal FHVP, when compared to ≤ 3 cm from HVO.

**Table 4:** Factors associated with optimal FHVP.

Factor	Univariate analysis		Multivariable analysis	
	OR (95% CI)	*p* value	OR (95% CI)	*p* value
Catheter tip distance from HVO				
≤ 3 cm (reference)	1	–	1	–
> 3 to ≤ 5 cm	1.69 (0.56–5.06)	0.349	1.78 (0.58–5.50)	0.316
> 5 to ≤ 8 cm	0.15 (0.05–0.42)	< 0.001	0.16 (0.05–0.47)	0.001
> 8 cm	0.12 (0.04–0.34)	0.001	0.14 (0.04–0.47)	0.002
History of HCC	0.53 (0.27–1.07)	0.075	0.47 (0.20–1.07)	0.072
Hepatic vein access				
RHV (reference)	1	–	1	–
MHV	1.53 (0.81–2.89)	0.194	1.50 (0.70–3.21)	0.295
Repeat HVPG (vs. initial)	1.46 (0.85–2.52)	0.171	1.34 (0.70–2.55)	0.377
ALP	1.004 (1.000–1.007)	0.040	1.003 (0.999–1.006)	0.177

Continuous data are presented as median (interquartile range) and categorical data as count (%).

ALP, alkaline phosphatase; CI, confidence interval; FHVP, free hepatic vein pressure; HCC, hepatocellular carcinoma; HVO, hepatic vein ostium; HVPG, hepatic venous pressure gradient; MHV, middle hepatic vein; OR, odds ratio; RHV, right hepatic vein.

### Clinical correlation and prognostication of HVPG

After excluding 14 patients, the baseline HVPGs of the remaining 214 patients were audited for clinical correlation and prognostication (Fig. 1). The HVPG of 127 patients (59.3%) had optimal FHVP while the remaining 87 patients (40.7%) had suboptimal FHVP. Two-hundred eight patients (97.2%) in this smaller cohort had one or more sign(s) of CSPH, where the difference between the two groups was not statistically significant (optimal FHVP vs. suboptimal FHVP: 98.4% vs. 96.5%, *p* = 0.399). However, the median (IQR) HVPG for procedures with suboptimal FHVP was significantly lower than HVPGs with optimal FHVP [14 (11–18) vs. 16 (13–19) mmHg, *p* = 0.010].

Thirty-three patients had ciHVPG based on the comparison of CSPH to their minimum expected HVPG. The prevalence of ciHVPG was significantly higher in patients whose baseline HVPG had suboptimal FHVP compared to those with optimal FHVP (25.3% vs. 8.7%, *p* = 0.002) (Table 5). When stratified by aetiology, 14 out of 76 patients with NASH (18.4%) had ciHVPG and this was not significantly different when compared to the 19 out of 137 patients (13.9%) without NASH who had ciHVPG (*p* = 0.430). Multivariable analysis showed that suboptimal FHVP was the only independent predictor of ciHVPG [OR 3.06 (95% CI 1.38–6.78), *p* = 0.006] (Model 1, Table 6). When NASH as a covariate was forced into the multivariable binary logistic regression, the significance of suboptimal FHVP as the sole predictor of ciHVPG was unaffected [OR 3.01 (95% CI 1.36–6.66), *p* = 0.007] (Model 2, Table 6).

**Table 5:** Comparison of ciHVPG among patients with optimal and suboptimal FHVP in their baseline HVPG.

Clinical signs of portal hypertension	Optimal FHVP (n = 127)	Suboptimal FHVP (n = 87)	*p* value
Ascites and/or hepatic hydrothorax, n (%)	1 (0.8)	2 (2.3)	0.568
Abdominal portosystemic collaterals on imaging^a^, n (%)	1 (0.8)	3 (3.5)	0.306
Gastric varices, n (%)	2 (1.6)	2 (2.3)	1.000
Low risk oesophageal varices, n (%)	0 (0.0)	4 (4.6)	0.026
High risk oesophageal varices, n (%)	5 (3.9)	6 (6.9)	0.360
History of oesophageal variceal bleed, n (%)	2 (1.6)	5 (5.8)	0.123
Total, n (%)	11 (8.7)	22 (25.3)	0.002

^a^In patients without gastroesophageal varices, ascites or hepatic hydrothorax.

ciHVPG, clinically incongruent hepatic venous pressure gradient; FHVP, free hepatic vein pressure; HVPG, hepatic venous pressure gradient.

**Table 6:** Factors associated with ciHVPG.

Factor	Univariate analysis		Multivariable analysis	
	OR (95% CI)	*p* value	OR (95% CI)	*p* value
Model 1 (Optimal multivariable logistic regression)				
Suboptimal FHVP	3.05 (1.41–6.59)	0.005	3.06 (1.38–6.78)	0.006
MELD score ≥ 15	0.18 (0.02–1.36)	0.096	0.29 (0.04–2.35)	0.249
Male gender	2.31 (1.02–5.24)	0.045	2.36 (0.99–5.53)	0.050
Albumin	1.07 (1.01–1.14)	0.029	1.06 (0.99–1.13)	0.101
Model 2 (NASH forced into multivariable logistic regression)				
Suboptimal FHVP	3.05 (1.41–6.59)	0.005	3.01 (1.35–6.67)	0.007
MELD score ≥ 15	0.18 (0.02–1.36)	0.096	0.302 (0.04–2.45)	0.248
Male gender	2.31 (1.02–5.24)	0.045	2.42 (0.99–5.71)	0.052
Albumin	1.07 (1.01–1.14)	0.029	1.05 (0.98–1.12)	0.146
NASH	1.42 (0.66–3.01)	0.369	1.31 (0.58–2.99)	0.689

CI, confidence interval; ciHVPG, clinically incongruent hepatic venous pressure gradient; FHVP, free hepatic vein pressure; FHVP, free hepatic vein pressure; MELD-Na, Model for End-Stage Liver Disease-Sodium; OR, odds ratio.

**Figure 4:**
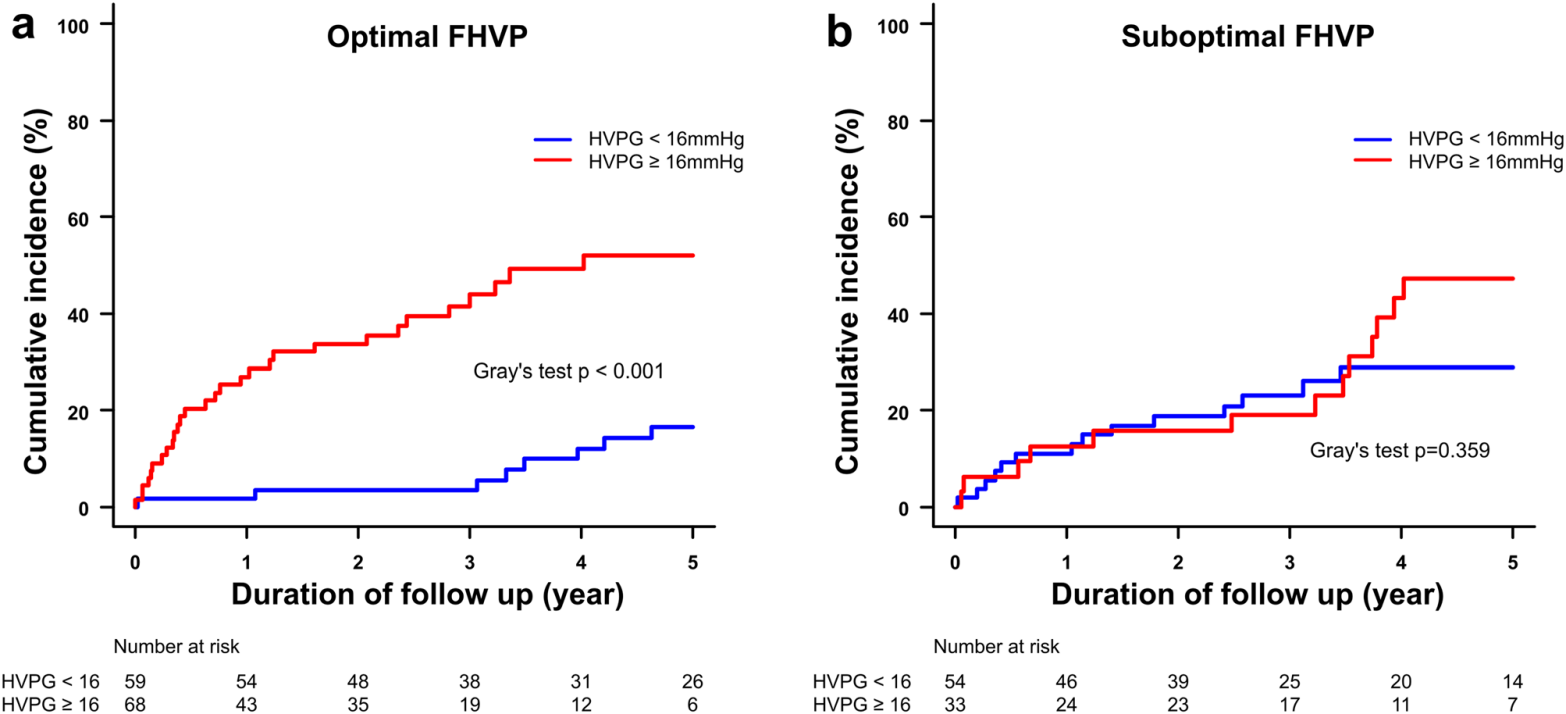
Cumulative incidence for the composite endpoints of cirrhosis-related death and liver transplantation for end-stage liver disease in patients based on their baseline hepatic venous pressure gradient, grouped according to (**a**) optimal FHVP and (**b**) suboptimal FHVP. FHVP, free hepatic vein pressure.

After a median follow-up of 34.8 (IQR 13.4–59.8) months, 67 patients died due to cirrhosis and six patients underwent liver transplantation for ESLD (Fig. 1). The overall 5-year cumulative incidence of the composite endpoints in the entire cohort was 35.6% (95% CI 28.4–42.8). Among patients with optimal FHVP, HVPG ≥ 16 mmHg was associated with a significantly higher cumulative incidence of cirrhosis-related death or liver transplantation for ESLD compared to HVPG < 16 mmHg [52.0% (95% CI 37.0–65.0) vs. 17.6% (95% CI 7.6–28.6), *p* < 0.001] (Fig. 4a). However, the association of HVPG ≥ 16 mmHg with the composite endpoints was lost in the other group of patients with suboptimal FHVP (*p* = 0.359) (Fig. 4b).

## Discussion

HVPG is the ‘gold standard’ in the assessment of PH in cirrhosis. However, the accuracy of HVPG requires strict adherence to quality standards during pressure measurements^1^. In a recent meta-analysis of individual patient data from the placebo or untreated arm of randomized controlled trials, the multicentre nature of the included studies was independently associated with poor HVPG reliability^27^. Although not addressed by the meta-analysis, it may be due to the contribution of poorer quality HVPGs performed in lower volume, non-academic centres. Thus, it is imperative that healthcare providers in centres providing care to patients with PH ensure that HVPG measurements are consistent, reliable and comparable across the entire spectrum of expertise, regardless of whether they are performed in academic or non-academic centres.

In our study, we sought to evaluate the effect of pressure measurements at different positions in the hepatic vein on the validity of HVPG and its impact on clinical care. We found that WHVP remained unchanged regardless of where the pressures were recorded. This is not surprising as the reading of WHVP reflects the hepatic sinusoidal pressure when the continuous column of fluid between the inflated balloon and hepatic sinusoids is formed^1^, independent of the catheter position. However, FHVP progressively increased when we measured the pressure at increasing distance from HVO. Rössle et al.^11^ showed that most of the hepatic veins assessed during HVPG were conical in shape and their diameter progressively decreased from proximal to distal. Therefore, when FHVP was recorded more distally in our study, it was likely that there was relative obstruction or progressive wedging of the narrower hepatic vein by the catheter, resulting in spurious elevation of the measured FHVP. The findings of our study showed that this false elevation of FHVP has major consequences on the accuracy and validity of HVPG. As IVCP was unaffected by the change in catheter position, FHVP-IVCP increased when measurements were taken in in a proximal to distal position in the hepatic vein. This resulted in violation of one important quality criterion of maintaining FHVP-IVCP ≤ 2 mmHg in a large majority of HVPG procedures when measurements were taken distally. Not surprisingly, the HVPG measured in this scenario progressively decreased because of an increasing FHVP and an unchanged WHVP. The calculated HVPG when FHVP-IVCP > 2 mmHg would inevitably be lower than its actual value, rendering it invalid as it underestimates the true portosystemic gradient.

In agreement with previous recommendation^1^, our study supports the recommendation that FHVP-IVCP > 2 mmHg can be reliably used as an important clue to an inaccurate HVPG. This should prompt the operator to repeat pressure measurements at a more proximal location in the HV, in addition to excluding hepatic vein outflow obstruction. In our study, catheter tip distance from HVO was independently associated with not achieving optimal FHVP only when it was > 5 cm. Additionally, we also showed that HVPGs measured at > 3 to ≤ 5 cm were not significantly different to those measured at ≤ 3 cm from HVO, implying that that the measurement of FHVP need not be performed strictly at 2–3 cm from HVO as previously recommended by various national, international and professional bodies^1,12,28–30^ but can be executed slightly more distally at ≤ 5 cm from HVO. In our experience, when the catheter is placed very proximally in the hepatic vein, this position is unstable and frequently results in prolapse of the catheter out of the hepatic vein into the inferior vena cava, especially during inflation of the balloon. By accepting a less stringent standard of pressure measurements at ≤ 5 cm instead of strictly at 2–3 cm from HVO, it may allow less experienced operators, especially in non-academic centres, to obtain a stable catheter position for pressure measurements without compromising the reliability and accuracy of HVPG.

We further analysed the impact of FHVP-IVCP on the clinical correlation of HVPG to the signs of CSPH and prognostication of survival in a smaller cohort of patients. After excluding patients on NSBB and occlusive portal vein thrombosis, the baseline HVPG of patients where the pressure measurements had suboptimal FHVP was significantly lower compared to those with optimal FHVP. The difference in HVPG between the groups was likely a reflection of the differences in catheter position during pressure measurement as there was no difference in the baseline demographics, complications of liver disease and markers of disease severity (Supplementary Table S1). Although the concept of ciHVPG has never been described before, it is based on well-defined HVPG thresholds for the development of clinical portal hypertension. When the measured HVPG is lower than expected for the clinical signs of portal hypertension in the patients, then we regarded the pressure measurements as ciHVPG. For example, it is well-established that gastroesophageal varices form when HVPG ≥ 10 mmHg^18^ but oesophageal varices only bleed when HVPG ≥ 12 mmHg^23^. Therefore, a hypothetical HVPG of 8 mmHg in a patient with small varices that had not bled and HVPG of 10 mmHg in another patient who had recent oesophageal variceal bleed were likely inaccurate and therefore classified as ciHVPG. Despite an overwhelming majority of patients (> 95%) having signs of CSPH in this smaller cohort, a quarter of patients with suboptimal FHVP had ciHVPG compared to 1 in 10 of patients with optimal FHVP. On multivariable analysis suboptimal FHVP was the only independent factor associated with ciHVPG. Interestingly, we also found that male gender had a higher odds of ciHVPG although the p value was just outside the pre-defined threshold for statistical significance. We believe that this was likely a type I error, as to the best of our knowledge, gender is not known to have any meaningful effect on pressure measurements during HVPG.

In terms of prognostication, HVPG ≥ 16 mmHg was strongly associated with the composite endpoints of cirrhosis-related death and liver transplantation for ESLD only when FHVP was optimal but not when it was suboptimal. In keeping with the findings from other studies^23,31^, the outcome of patients with accurately measured HVPG < 16 mmHg in our study was good, reaffirming its role as a prognostic tool. The poor correlation of HVPG to CSPH and loss of its prognostic value in patients with suboptimal FHVP in our study were likely due to HVPGs in these patients underestimating the actual portosystemic gradient. This resulted in misclassification of some patients with actual HVPG ≥ 10–12 mmHg as < 10–12 mmHg and ≥ 16 mmHg as < 16 mmHg. Therefore, disproportionately more patients with suboptimal FHVP had ciHVPG compared to those with optimal FHVP. The misclassification of higher risk patients (HVPG ≥ 16 mmHg) into the lower risk group (HVPG < 16 mmHg) led to the cumulative incidence of the composite endpoints in both groups to converge, resulting in loss of statistically significant difference between HVPG ≥ 16 mmHg and < 16 mmHg, diminishing its prognostic value.

In our study, almost 10% of patients with accurate HVPG, in the group of patients with optimal FHVP, had ciHVPG. It may be possible that some of these patients had porto-sinusoidal vascular disease, a condition with lower HVPG but often misdiagnosed as cirrhosis^32^, as we do not routinely perform transient elastography^33^ or liver biopsy^34^ in patients already diagnosed with cirrhosis based on clinical, biochemical, sonographic and/or endoscopic findings. Secondly, it is now well described that about 10% of patients with cirrhosis due to NASH can develop signs of portal hypertension or decompensation at HVPG < 10–12 mmHg^35,36^. In our study, the prevalence of ciHVPG was numerically higher in patients with NASH compared to non-NASH but the difference was not statistically significant. When NASH as a variable was forced into the multivariable logistic regression model, it also did not emerge as an independent predictor of ciHVPG. This difference between our study and the others may be due to the much smaller number of patients with NASH cirrhosis in our study (n = 79) compared to the studies by Sanyal et al.^35^ (n = 258) and Bassegoda et al.^36^ (n = 548). Primary biliary cholangitis (PBC) is another well-described chronic liver disease with a prominent pre-sinusoidal component of portal hypertension and HVPG may also underestimate the actual portal pressure gradient^37^. We were unable to verify this in our study as the number of patients with PBC was very small (n = 10). Finally, in a study from India, 10.3% of patients with decompensated cirrhosis predominantly due to alcohol use had HVPG < 10 mmHg^38^. This finding concurs with ours and suggests that approximately 1 in every 10 patients with cirrhosis, not necessarily due to NASH, and presenting with either definite signs of CSPH or clinical decompensation, may have HVPG lower than the traditionally accepted threshold^18,19,23^. This finding is novel, but needs to be validated in larger studies from other centres.

We would like to acknowledge the limitations of this study. Our study was a single centre series hampered by retrospective analysis. Furthermore, there is no standard method of measuring catheter tip distance from HVO during HVPG, either intra-procedurally or during review of fluoroscopy images, although rater agreement was good to excellent in our study. However, this method of measuring catheter tip distance from HVO relies on static images captured during fluoroscopic projections during HVPG which may not be reflective of the actual procedures in real-time, including the process of WHVP and FHVP measurements. Furthermore, the quality of the images was not standardised, and this may also limit the accuracy of the measured distance. Secondly, our study would not account for the pressure changes due to individual patient variation in the caliber and shape of the hepatic veins as this would require haemodynamic assessments, including FHVP, to be repeated in the same vein of the same patient at different distance from HVO^11^. This was not assessed in our retrospective study as such repeat pressure measurements in the same patient is not routinely performed in clinical practice. Moreover, the results of our study may need to be interpreted with some caution, as the routine use of 5.5 F catheters in our practice likely allowed measurement of FHVP more distally in the hepatic veins with less relative obstruction or wedging of the veins^11^ compared to larger 7-8F catheters commonly used in others centres^9,10,13^. Finally, pressure measurement tracings are not permanently recorded in our centre and therefore, were not available for review in this study. As such, we were not able to verify the accuracy of the pressure measurements, especially for WHVP, which requires interpretation of pressure tracings after an adequate period of stabilisation. This may further introduce uncertainties into the findings and conclusions of our study.

## Conclusion

HVPG likely decreases when measured at progressively farther distance from HVO due to its effect on increasing FHVP. HVPGs measured within 5 cm from HVO may be more likely to comply with the quality criterion of FHVP-IVCP ≤ 2 mmHg but pressure measurements beyond this threshold may correlate more poorly with signs of CSPH and lose its prognostic value in cirrhosis. However, due to the limitations of our study, these findings need to be verified in larger prospective studies.

### Supplementary Information

Supplementary Table S1.

## Data Availability

The datasets used during the current study are available from the corresponding author on reasonable request.

## Abbreviations

PBC: Primary biliary cholangitis
CI: Confidence interval
CSPH: Clinically significant portal hypertension
ESLD: End-stage liver disease
FHVP: Free hepatic vein pressure
FHVP-IVCP: Difference between free hepatic vein pressure and inferior vena cava pressure at the level of hepatic vein
HVO: Hepatic vein ostium
HVPG: Hepatic venous pressure gradient
ICC: Intra-class correlation coefficient
IVCP: Inferior vena cava pressure at the level of hepatic vein
NASH: Nonalcoholic steatohepatitis
OR: Odds ratio
WHVP: Wedged hepatic vein pressure

## References

[1] Groszmann RJ, Wongcharatrawee S (2004). The hepatic venous pressure gradient: Anything worth doing should be done right. Hepatology.

[2] Myers J, Taylor W (1951). An estimation of portal venous pressure by occlusive catheterization of an hepatic venule. J. Clin. Invest..

[3] Viallet A (1975). Hemodynamic evaluation of patients with intrahepatic portal hypertension. Relationship between bleeding varices and the portohepatic gradient. Gastroenterology.

[4] Vinel JP, Cassigneul J, Levade M, Voigt JJ, Pascal JP (1986). Assessment of short-term prognosis after variceal bleeding in patients with alcoholic cirrhosis by early measurement of the portohepatic gradient. Hepatology.

[5] Boyer TD, Triger DR, Horisawa M, Redeker AG, Reynolds TB (1977). Direct transhepatic measurement of portal vein pressure using a thin needle. Comparison with the wedged hepatic vein pressure. Gastroenterology.

[6] Lin HC (1989). Comparison between portal vein pressure and wedged hepatic vein pressure in hepatitis B-related cirrhosis. J. Hepatol..

[7] Perelló A (1999). Wedged hepatic venous pressure adequately reflects portal pressure in hepatitis C virus-related cirrhosis. Hepatology.

[8] Groszmann RJ, Glickman M, Blei AT, Storer E, Conn HO (1979). Wedged and free hepatic venous pressure measured with a balloon catheter. Gastroenterology.

[9] La Mura V (2010). Right atrial pressure is not adequate to calculate portal pressure gradient in cirrhosis: A clinical-hemodynamic correlation study. Hepatology.

[10] Silva-Junior G (2015). The prognostic value of hepatic venous pressure gradient in patients with cirrhosis is highly dependent on the accuracy of the technique. Hepatology.

[11] Rössle M, Blanke P, Fritz B, Schultheiss M, Bettinger D (2016). Free hepatic vein pressure is not useful to calculate the portal pressure gradient in cirrhosis: A morphologic and hemodynamic study. J. Vasc. Interv. Radiol..

[12] Berzigotti A, Seijio S, Reverter E, Bosch J (2013). Assessing portal hypertension in liver diseases. Expert Rev. Gastroenterol. Hepatol..

[13] Ripoll C (2005). Influence of hepatic venous pressure gradient on the prediction of survival of patients with cirrhosis in the MELD era. Hepatology.

[14] Ministry of Health, Singapore. Table differentiating research from service evaluation, clinical audit, surveillance and outbreak investigations. https://www.moh.gov.sg/docs/librariesprovider5/legislation/table-differentiating-research-from-research-like-activities_31jan2018.pdf. Accessed 4 January 2021.

[15] Chang PE, Wong GW, Li JWQ, Lui HF, Chow WC, Tan CK (2015). Epidemiology and clinical evolution of liver cirrhosis in Singapore. Ann. Acad. Med. Singap..

[16] Noureddin M (2020). Attribution of nonalcoholic steatohepatitis as an etiology of cirrhosis for clinical trials eligibility: Recommendations from the multi-stakeholder Liver Forum. Gastroenterology.

[17] Lu Q (2021). Hepatic venous-portal gradient (HVPG) measurement: Pearls and pitfalls. Br. J. Radiol..

[18] Groszmann RJ (2005). Beta-blockers to prevent gastroesophageal varices in patients with cirrhosis. N. Engl. J. Med..

[19] Bosch J (1980). Hepatic hemodynamics and the renin-angiotensin-aldosterone system in cirrhosis. Gastroenterology.

[20] Vilgrain V, Lebrec D, Menu Y, Scherrer A, Nahum H (1990). Comparison between ultrasonographic signs and the degree of portal hypertension in patients with cirrhosis. Gastrointest. Radiol..

[21] Merli M (2003). Incidence and natural history of small esophageal varices in cirrhotic patients. J. Hepatol..

[22] North Italian Endoscopic Club for the Study and Treatment of Esophageal Varices (1988). Prediction of the first variceal hemorrhage in patients with cirrhosis of the liver and esophageal varices. A prospective multicenter study. N. Engl. J. Med..

[23] Garcia-Tsao G, Groszmann RJ, Fisher RL, Conn HO, Atterbury CE, Glickmn M (1985). Portal pressure, presence of gastroesophageal varices and variceal bleeding. Hepatology.

[24] Berzigotti A (2011). Prognostic value of a single HVPG measurement and Doppler-ultrasound evaluation in patients with cirrhosis and portal hypertension. J. Gastroenterol..

[25] Tan HK (2021). Poor outcomes of cirrhosis due to nonalcoholic steatohepatitis compared with hepatitis B after decompensation with ascites. Am. J. Gastroenterol..

[26] Kanda Y (2013). Investigation of the freely available easy-to-use software ‘EZR’ for medical statistics. Bone Marrow Transplant..

[27] Bai W, Al-Karaghouli M, Stach J, Sung S, Matheson GJ, Abraldes JG (2021). Test-retest reliability and consistency of HVPG and impact on trial design: A study in 289 patients from 20 randomized controlled trials. Hepatology.

[28] Reiberger T, Schwabl P, Trauner M, Peck-Radosavljevic M, Mandorfer M (2020). Measurement of the hepatic venous pressure gradient and transjugular liver biopsy. J. Vis. Exp..

[29] Mandorer M (2023). Austrian consensus on the diagnosis and management of portal hypertension in advanced chronic liver disease (Billroth IV). Wien Klin Wochenschr.

[30] de Francis R, Bosch J, Garcia-Tsao G, Reiberger T, Ripoll C, Baveno VII Faculty (2022). Baveno VII—Renewing consensus in portal hypertension. J. Hepatol..

[31] Patch D (1999). Single portal pressure measurement predicts survival in cirrhotic patients with recent variceal bleeding. Gut.

[32] Seijio S (2012). Role of hepatic vein catheterisation and transient elastography in the diagnosis of idiopathic portal hypertension. Dig. Liv. Dis..

[33] Elkrief L (2021). Liver stiffness by transient elastography to detect porto-sinusoidal vascular disease with portal hypertension. Hepatology.

[34] De Gottardi A (2019). Porto-sinusoidal vascular disease: proposal and description of a novel entity. Lancet Gastroenterol. Hepatol..

[35] Sanyal AJ (2019). The natural history of advanced fibrosis due to nonalcoholic steatohepatitis: Data from the simtuzumab trials. Hepatology.

[36] Bassegoda O (2022). Decompensation in advanced nonalcoholic fatty liver disease may occur at lower hepatic venous pressure gradient levels than in patients with viral disease. Clin. Gastroenterol. Hepatol..

[37] Navasa M (1987). Portal hypertension in primary biliary cirrhosis. Relationship with histological features. J. Hepatol..

[38] Jindal A, Sarin SK (2019). Influence of HVPG on clinical outcomes in decompensated cirrhosis: need to redefine clinically significant portal hypertension. Hepatology.

