# Supplementary material for: Impact of catheter tip to hepatic vein ostium distance on the validity and prognostication of hepatic venous pressure gradient in cirrhosis
Source: Sci Rep. 2023 Oct 9;13:16980. doi: 10.1038/s41598-023-44016-7 (PMC10562361; doi:10.1038/s41598-023-44016-7)
Supplement: Supplementary file 1 — Supplementary Table S1. [file 41598_2023_44016_MOESM1_ESM.docx]

**Supplementary Table S1. Baseline characteristics of patients selected for clinical outcomes audit**

| **Characteristics** | **Optimal FHVP (n=127)** | **Suboptimal FHVP (n=87)** | **p value** |
| --- | --- | --- | --- |
| Age, years | 62.1 (55.1-68.5) | 63.2 (53.5-68.0) | 0.988 |
| Gender, n (%)  Male  Female | 75 (58.3)  53 (41.7) | 47 (54.0)  40 (46.0) | 0.576 |
| Aetiology of cirrhosis, n (%)  Viral hepatitis  Alcoholic liver disease  NASH/cryptogenic  Autoimmune liver disease  Others | 34 (26.8)  20 (15.7)  48 (37.8)  10 (7.9)  15 (11.8) | 28 (32.2)  8 (9.2)  39 (44.8)  7 (8.0)  5 (5.7) | 0.300 |
| Clinical signs of portal hypertension, n (%)  Abdominal portosystemic collaterals  History of variceal bleed  Non-bleeding varices  Ascites/Hepatic hydrothorax  None | 71 (55.9)  52 (40.9)  61 (48.0)  48 (37.8)  2 (1.6) | 48 (55.2)  36 (41.4)  45 (51.7)  30 (34.5)  3 (3.5) | 1.000  1.000  0.677  0.666  0.399 |
| Other complications, n (%)  Hepatic encephalopathy  Hepatocellular carcinoma | 6 (4.7)  14 (11.0) | 2 (2.3)  15 (17.2) | 0.477  0.244 |
| Haemoglobin (g/dL) | 10.6 (9.3-12.1) | 10.4 (9.1-12.6) | 0.841 |
| WBC (x10^9^/L) | 4.84 (3.80-6.87) | 4.84 (3.79-6.27) | 0.697 |
| Platelet (x10^9^/L) | 100 (74-140) | 106 (80-147) | 0.456 |
| Albumin (g/L) | 32 (27-3) | 33 (28-37) | 0.430 |
| Bilirubin (µmol/L) | 21 (15-38) | 22 (15-36) | 0.705 |
| ALT (U/L) | 33 (22-51) | 33 (22-48) | 0.939 |
| AST (U/L) | 51 (37-70) | 46 (37-65) | 0.518 |
| INR | 1.14 (1.06-1.23) | 1.12 (1.02-1.24) | 0.188 |
| Urea (mmol/L) | 4.4 (2.9-6.1) | 4.5 (3.5-5.6) | 0.618 |
| Na (mmol/L) | 137 (135-139) | 137 (134-139) | 0.930 |
| Creatinine (µmol/L) | 68 (56-88) | 66 (53-81) | 0.517 |
| MELD score | 9 (8-13) | 9 (8-12) | 0.238 |
| Child-Pugh score | 6 (5-8) | 6 (5-8) | 0.674 |
| Continuous data are presented as median (interquartile range) and categorical data as count (%)  ALP, alkaline phosphatase; ALT, alanine aminotransferase; AST, aspartate aminotransferase; INR, international normalized ratio; MELD , Model for End-Stage Liver Disease; WBC, white blood cell | | | |
